# RAS/RAF/MEK/MAPK signaling pathway as a therapeutic target in breast cancer: Emphasis on a novel carrier for tamoxifen and digestion behaviors

**DOI:** 10.22038/ajp.2024.25253

**Published:** 2025

**Authors:** Niloofar Mansouri, Melika Daneshgar, Farzaneh Khojasteh, Zahra Modaresi, Reza Taheri, Parisa Mokaberi, Mohammad Reza Saberi, Jamshidkhan Chamani

**Affiliations:** 1 *Department of Biology, Mashhad Branch, Islamic Azad University, Mashhad, Iran*; 2 *Department* *of* *Medicinal* *Chemistry,* *School* *of* *Pharmacy,* *Mashhad* *University* *of* *Medical* *Sciences,* *Mashhad,* *Iran*

**Keywords:** Cellulose nanocrystals Tamoxifen delivery XRD, TEM, Breast cancer treatment RAS/RAF/MEK/MAPK signaling

## Abstract

**Objective:**

This research attempted to increase the bioactivity and solubility and reduce the side effects of Tamoxifen (TMX) by using the cellulose nanocrystals (CNCs) extracted from walnut shells as a carrier and studied the interaction behavior of CNCs-TMX with hemoglobin.

**Materials and Methods:**

The synthesized CNCs and CNCs-TMX were analyzed through the usage of XRD, FTIR, TEM, SEM, and multi-spectroscopic techniques. A real-time PCR assay was also conducted to further unravel the underlying mechanism of CNCs- TMX.

**Results:**

Our synthesized products including CNCs and CNCs- TMX had spherical morphologies in small sizes of 17.42 nm and 56.38 nm, respectively. The changes in FTIR spectrum signified the induced alterations in the samples functional group during the steps of preparation, while the crystallinity index of CNCs was 71.35%. Fluorescence spectroscopy confirmed the quencher functionality of CNCs-TMX along with the dominance of static quenching mechanism. Also, synchronous fluorescence displayed its binding to Hb in the vicinity of Tryptophanresidue. FRET was applied to calculate the interaction energy transfer of 0.18 nm. Next to achieving satisfactory results from oxygen-hemoglobin dissociation studies, the presence of CNCs-TMX caused a reduction in hemoglobin affinity for oxygen.

**Conclusion:**

Our findings pointed out the remarkable potential of TMX-loaded CNCs, derived from walnut shell, in suppressing the proliferation, migration, and invasion of breast cancer cells by quelling the RAS/RAF/MEK/MAPK signaling pathways. The gathered data approved the promising applicability of the obtained CNCs from walnut shell in the delivery system of anti-cancer drugs throughout pharmaceutical applications.

## Introduction

**Figure 1:**
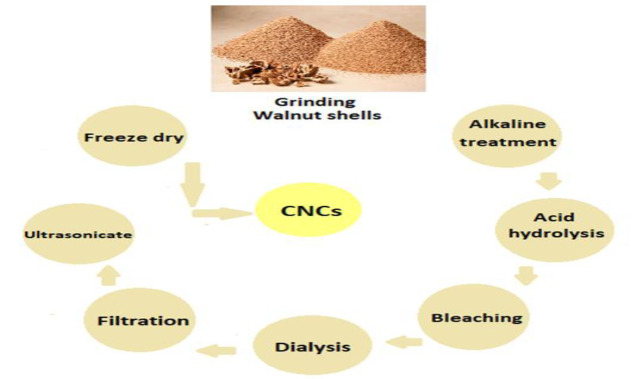
Graphical presentation of the cellulose nanocrystals synthesis steps

The last decade implicated the emergence of nanoparticle-based therapeutic systems as promising approaches for the treatment of cancer. The selective delivery of anti-cancer drugs is a necessity for the removal of tumors and providing a better prognosis(Levi 1999; Pourmobini et al. 2021; Zhang et al. 2020).The unique nanomaterials of cellulose nanocrystals (CNCs) are abundant and inexhaustible which are usually obtained from acid hydrolysis at high temperatures (Figure 1) (Phanthong et al. 2018; Yu et al. 2021). Their crystalline nature is initially procured from natural cellulose fibers that are provided from renewable sources and removal of amorphous acidic parts (Kumar et al. 2014; Olad, Doustdar and Gharekhani 2020). Next to degradability and renewability as the most important features of CNCs (Seabra et al. 2018), they are also known for their optical, mechanical, rheological, and chemical properties (Rao et al. 2007; Zheng et al. 2019).

**Figure 2:**
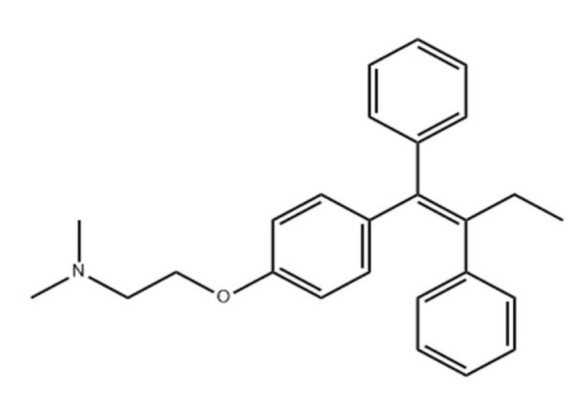
The chemical structure of tamoxifen

Tamoxifen (TMX) (Figure 2) can function as an anti-cancer medicine that exploits estrogen to cure all stages of breast cancer by hormones in the cases of men and women (Osborne 1998), and TMX can be used after surgery to reduce the risk of breast cancer at the early stage of disease TMX blocks the effects of estrogen on hormone receptor-positive breast cancercells by binding to estrogen receptors on cells and prevent the attachment of estrogen to the cancer cell, which stops the cell dose of estrogen signals for growth and proliferation; however, this medicine has no effect the cases of breast cancer with negative hormone receptors (Jaiyesimi et al. 1995; Jordan 1994; Rajabi et al. 2024).

Breast cancer is a condition characterized by the uncontrolled proliferation and growth of breast cells that originate from the ducts or lobules and can spread to other parts of the body(Feng et al. 2018). The majority of new cases include women over the age of 50, the risk of fatality from breast cancer by the age of 85 is estimated to be 1 in 77 individuals(Giaquinto et al. 2022).

The aim of the present study wasto produces CNCs from walnut shell as a cheap and biocompatible alternative platform for the delivery of TMX. In the section, we characterized the extracted CNCs and CNCs-TMX by using Dynamic light scattering to determine the particle size and zeta potential, X-ray diffraction(XRD)to configure the crystallinity index, Fourier transform infrared (FTIR) to investigate the chemical structure, and Field emission scanning electron microscopy (FE-SEM) and transmission electron microscopy (TEM)to distinguish their shape and morphology (Mohamed et al. 2015), which was followed by investigating the interaction between CNCs-TMX and human hemoglobin protein. The technique of fluorescence spectroscopy was included to detect synchronous fluorescence, Resonance light scattering (RLS), circular dichroism (CD), Forster resonance energy transfer (FRET), Three-dimensional fluorescence (3D) fluorescence. On the other hand, our study aimed to conduct *in vitro *investigations on the potential of TMX-loaded CNCs, derived from walnut shells, in inhibiting the growth and metastasis of two distinct types of breast cancer cells (SkBr3 and T-47D). Additionally, we sought to uncover the molecular mechanisms underlying the impact of CNCs-TMX on RAS/RAF/MEK/MAPK signaling pathways.

## Materials and Methods

Materials

Walnut shells were purchased from a grocery store in Mashhad, Iran. The exerted chemical reagents, including sodium hydroxide %5 (NaOH), sodium chlorite (NaClO2), acetic acid (CH3COOH), sodium bicarbonate %2, ethylene diamine tetra acetic acid (EDTA %0.05), and sulfuric acid 64% (H2SO4) was purchased from Merck, Germany. In addition, dialysis membrane was procured from Sigma- Aldrich, USA and ethanol %20 was obtained from Pars Toos, Iran. Next to attaining TMX from Sigma-Aldrich, USA, we purchased hemoglobin protein and potassium dihydrogen phosphate from Sigma-Aldrich, USA while breast cancer cell lines (SkBr3 and T-47D) were acquired from Pasture institute (Tehran, Iran). All the other applied chemicals and reagents in our experiment were of analytical grade.

Method

**Extraction of CNCs**

The walnut shells were initially pretreated to isolate the nano-cellulose, through the described method in the work of (Rodrigues, Venâncio and Lima 2012). The walnut shells were ground and sieved through 60 mesh (0.25 mm) to be dried in an oven at 105 °C and stored in a desiccator. The powdered sample was placed on a heater with 5% sodium hydroxide and deionized water for 2 hr. Then, the cooled samples were poured into the falcon to be centrifuged for 3 times (around 6000 rpmfor8 min each time). The topping liquid of the falcons was removed to place the sediments in a laboratory oven at a temperature of 80°C for drying. Once the sample alkalized with 64% sulfuric acid was placed on the heater for 90 min, the cooled sample was poured into the falcons to be centrifuged for 3 times (8000 rpm for 10 min) up to the point of completely removing its acidic smell. These sediments were required to be dried in an oven. Two separate beakers were used to pour the acidified sample with sodium chlorite and 5% sodium hydroxide along with acetic acid and distilled water. Thereafter, the beakers were placed on the heater for 4 hr to be subsequently centrifuged twice at 10000 rpm for 5 min at each round. Thereafter, the sediments obtained from the first bleaching were placed on the heater for 90 min with the same amounts of the obtained chemicals at the first stage up to achieving white sediments which were then centrifuged for two rounds (8000 rpm for 10 minutes) The resultant was poured into the dialysis membrane along with small portions of the existing liquid at the bottom of falcons for 15 hr. The stirring was continued to complete the dialyzing process and separate the impurities. The produced liquid was filtered 3 times by a syringe filter to detach the larger sized materials and impurities. The filtered liquid was poured into a falcon or a microtube to be sonicated for 15 min in order to reduce the size of particles and disperse the nanoparticles of the liquid. At this point, we considered the application of an Adeline 35 KHz device, made in Germany. Finally, the resulting sediments were dried by a freeze-dry machine at a very low temperature to be used in the characterization process along with CNCs powder. Our research also implicated the use ofAlpha 1-4 L plus machine, Marti Christ, made in Germany.

Preparation of CNCs-TMX

The CNCs solution was passed through a syringe filter, poured into a falcon with a pre-weighed amount of TMX, and shaken to be thoroughly mixed in order to be placed again in the sonicator device.

Drug loading capacity (LC) and encapsulation efficiency (EE) measurement

UV-Vis spectroscopy was used to measure the loading capacity and encapsulation efficiency of TMX-loaded CNCs. Once the TMX-loaded CNCs dispersed in distilled water were thoroughly mixed, the concentration of TMX was measured by a UV-Vis spectrophotometer at the wavelength of 271 nm. To prepare the calibration curve, several standards of TMX at the2.7 × 10^6 µM concentration were prepared in distilled water, which was continued by calculating the Loading Capacity and Encapsulation Efficiency through the following equations:

Lc%=((Initial drug amountin formulation (mg)- Unentrapped drug (mg))Total weigh to f sample mg×100

Eq1

$\mathrm{Encapsulation Efficiency}\left(\%\right)=\frac{\left((\mathrm{Initial drug amountin formulation}(\mathrm{mg})-\mathrm{Unentrapped drug}(\mathrm{mg})\right)}{\mathrm{Initial drug amount in formulation}(\mathrm{mg})\times 100}$

Eq2

Interaction of CNCs-TMX with hemoglobin protein

The hemoglobin protein was dissolved in the potassium dihydrogen phosphate buffer and placed on the heater to achieve a solution color of brown as a sign of dissolved hemoglobin in the buffer, while the CNCs-TMX was liquefied in deionized water. At this point, the protein was positioned in the cell to face the prepared solution, which was proceeded by simultaneously measuring the emission fluorescence in a fluorescence device at ∆λ

= 15 and ∆λ = 60 wavelengths, along with RLS.

Characterization Zeta potential

Particle size, polydispersity (PDI), and potential alterations were determined through the applied dynamic light scattering by a Zeta-sizer nano-zs90 instrument (Malvern Instruments, Worcestershire, UK). All the measurements were recorded at 25°C and the fixed angle of 173°,while the count rate of 100–200 kilocounts per second (KCPS) was maintained by diluting the samples with deionized water at pH 7. We performed three replicate measurements per sample.

**TEM**

TEM experiments were conducted to investigate the fine morphologies and porous structure of CNCs. Their nanostructure was analyzed by the utilization of a JEOL 2000 EX II electron microscope at an accel- Erating voltage of 120 KV. In addition, Image j software was used to calculate the average diameter of CNCs and nanoparticles (Maaloul et al. 2021).

FE-SEM

The TMX-CNCs (Tamoxifen-CNC) sample was dried on a plasma-treated (glow-discharged) carbon-film grid to have its micromorphology determined by (JEOL JEM- 2100 Electron Microscope, Tokyo, Japan) at an accelerating voltage of 200 KV. FEI Quanta 250 FEG SEM was used to complete the SEM analysis. The images were acquired at a nominal magnification of 20,000. For the purpose of measuring the carbon and oxygen elements of hydrolyzed sample and composed CNCs on the basis of energy scattering, we exerted x-rays. Spectroscopy (EDX) at an accelerator voltage of 20 kV for 60 minutes.

XRD

We employed an X-ray diffractometer (Panalytical, Netherlands) with Cu-Kα radiation to determine the diffraction patterns and crystallinity index of our samples. The crystallinity (Xc) of different samples was calculated through Eq 3:

Crl= 𝐼002−𝐼𝑎𝑚 × 100 (1)

𝐼002

of 2 cm^-1^.Then, the obtained spectra were smoothened by the application of OMNIC 9.2.86 software9.2.86 software (Jiang et al. 2020).

Interaction with Hb

**Fluorescence** **spectroscopy** **measurements**

All of the fluorescence measurements were performed on an F-2500 spectrophotometer (Hitachi, Japan) that was equipped with 1.0 cm quartz cells and a xenon lamp light. The fluorescence intensities were adjusted for inner filter and dilution effects prior to processing the data (Shahabadi and Zendehcheshm 2020).

The synchronous fluorescence spectra were carried out through the simultaneous scanning of excitation and emission monochromators, which only displayed the Tryptophan and Tyrosin remainders of Hb in the wavelength spacing (∆λ) at 15 and 60 nm, respectively(Wang et al. 2022).

RLS spectra were registered by scanning both the excitation and emission monochromators through a common spectrofluorometer with ∆λ = 0 nm.

The three-dimensional spectra were provided by a Hitachi F- 2500 spectrofluorometer equipped with 1.0-cm quartz cells.

Circular dichroism

A quartz cell with a path length of 1mm was used for circular dichroism (CD). The required spectra were collected through a scanning speed of 100 nm/min, a bandwidth of 1nm, and a measuring range of 190-250 nm. Dry nitrogen was exerted to purge the machine before and during the tests.

FTIR

FTIR experiments were conducted to investigate the chemical structure of cellulose constituents, as well as CNCs isolated from walnut shells. The samples were loaded onto Potassium bromide (KBr) discs to record the spectral databy Equinix

55 spectrometer (m/s Thermo Fisher Scientific –instruments, USA) throughout the wavenumber range of 400-4000 cm^-1^ with an average of 32 scans at a resolution

FRET

To record the absorption spectra throughout the range of 200–700 nm at room temperature, a Jasco V-630 double- beam spectrophotometer was connected to a personal computer. The absorption spectra of all of the samples were obtained by using a 1 cm quartz cell.

Oxygen-Hemoglobin dissociation

Oxygen-hemoglobin dissociation curve represents the association of bounded oxygen to the amount of Hb and the partial pressure (PO2) of oxygen in blood, which is exhibited through a sigmoidal curve. The measurement is completed by a blood gas device with a silver anode/platinum cathode system in an electrolyte solution. Also, the mixture was detached from the solution by a semi-permeable membrane.

Cell culture

Cells (SkBr3 and T-47D cells) were maintained in RPMI 1640 medium consisted of 10% fetal bovine serum, 100 U/ml of penicillin, and 100 mg/L of streptomycin. All of the cultures with cancer cells were maintained at 37°C in a humidified atmosphere by 5% CO2 and 95% air.

Cytotoxicity assay

The cytotoxicity of CNCs-TMX was determined through (3-(4,5-Dimethylthiazol- 2-yl)-2,5-Diphenyltetrazolium Bromide) (MTT) assay(Öztürk et al. 2017). Accordingly, the cells were plated in 96- well plates and treated with various concentrations (0.002, 0.004, 0.006, 0.008

and 0.01 mg/ml) of CNCs-TMX for 24, 48, and 72 hr, respectively. Subsequent to being incubated for the selected time periods at 37°C in a humidified chamber, MTT reagent (5 mg/mL in phosphate buffered saline (PBS)) was added to each well to conduct another incubation for 4 hr. Then, the medium was discarded and formazan induced by metabolically viable cells was dissolved in 150 ml of dimethyl sulfoxide (DMSO). The absorbance was measured by an infinite M200 Pro microplate reader at the experimental wavelength of 570 nm.

Real-time PCR assay

Next to the performance of RT-PCR assayin accordance to previous descriptions (Xiang et al. 2015), we extracted total RNA by using TRIzol (Invitrogen, Carlsbad, CA, USA) and determined certain concentrations of RNA through the utilization of a Nano DROP 2000 Spectrophotometer (Thermo Scientific, USA). In addition, Primer Script TM First Strand cDNA Synthesis Kit was exerted to conduct Reverse transcription for the synthesis of cDNA. The conditions of PCR amplification implicated 40 cycles of 95°C for 30 sec, 95°C for 5 sec, and 60 °C for 30 sec. The usage of SYBR Premix Ex Taq helped in detecting the expression levels of pivotal genes in RAS/RAF/MEK signaling pathways.

***In vitro*** **release behavior**

We considered the usage of dialysis membrane to evaluate the release of TMX from CNCs-TMX, TMX-loaded Pluronic P-123 (PEG-PPG-PEG) micelles(Moin et al. 2021) and TMX-loaded niosomes (Akbarzadeh et al. 2022) in the cases of simulated stomach and small intestine conditions (Chang et al. 2017) 3.0 ml of CNCs-TMX, TMX-loaded PEG-PPG-PEG

micelles, or TMX-loaded niosomes were placed into a dialysis sac (12 kDa) and mixed with 3.0 mL of simulated gastric fluid (SGF) that contained HCl, NaCl, and pepsin (with concentration of 3.2 mg/mL) at a pH of 1.2. The bag was submerged in

150 mL of release medium, which was composed of enzyme-free SGF and ethanol, to be incubated on a stirrer at 37 °C for 2 hr. Then, the mixture was charged with 6 mL of a simulated intestinal fluid (SIF) that contained NaOH, monobasic potassium phosphate, and pancreatic (at a concentration of 10 mg/mL) with the pH value of 7.5. The dialysis bag was then placed into 150 mL of fresh release medium, consisted of enzyme-free simulated intestinal fluid (SIF) and also ethanol, to be incubated on a stirrer at 37°C for 4 hr. Moreover, the application of ethanol in the release medium helped in creating a sinking condition to overcome the low solubility of TMX in water. The aliquots of outer release medium were collected at different time intervals (1, 2, 3, 4, 5, and 6 hr) to determine the amount of released TMX and measure their absorbance at 235 nm, while the aliquots were returned to the release medium afterwards to keep the volume of medium constant. The concentration of released TMX was calculated through the TMX standard curves, which were prepared by the same release medium.

## Results

### Morphological analysis

#### Particle size, PDI, and ζ-potential

**Figure3:**
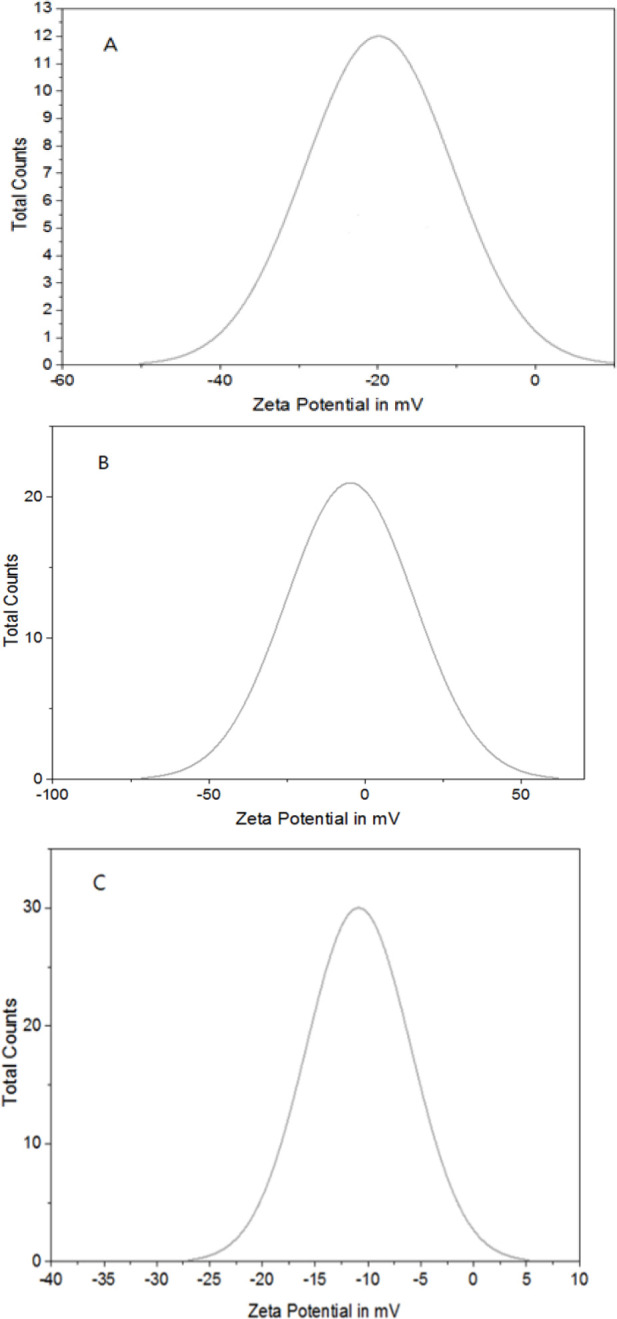
Zeta potential values of (A); CNCs, (B); TMX and (C); CNCs-TMX.

Figure3(A-C) displays the measured Zeta potential of CNCs, CNCs-TMX, and TMX to be -16.69, -4.74, and -10.88 mV, respectively. Meanwhile, the sizes of CNCs, CNCs-TMX, and TMX were also measured and reported to be 361.93 ±10.11 mV, 366.53± 4.72 mV, and 25.3±20.23 mV, respectively. The parameter of Polydispersity index (PDI) is applied to measure the size distribution of particles or molecules in a solution. A low PDI value indicates a narrow size distribution with a small variation in particle size. The calculated PDI values of CNCs and CNCs- TMX were 0.16 and 0.11, respectively. A narrow size distribution is desirable in many applications, especially in drug delivery systems, as it ensures the dominance of a consistent behavior (Prasanna and Mitra 2020).

#### TEM

**Figure 4:**
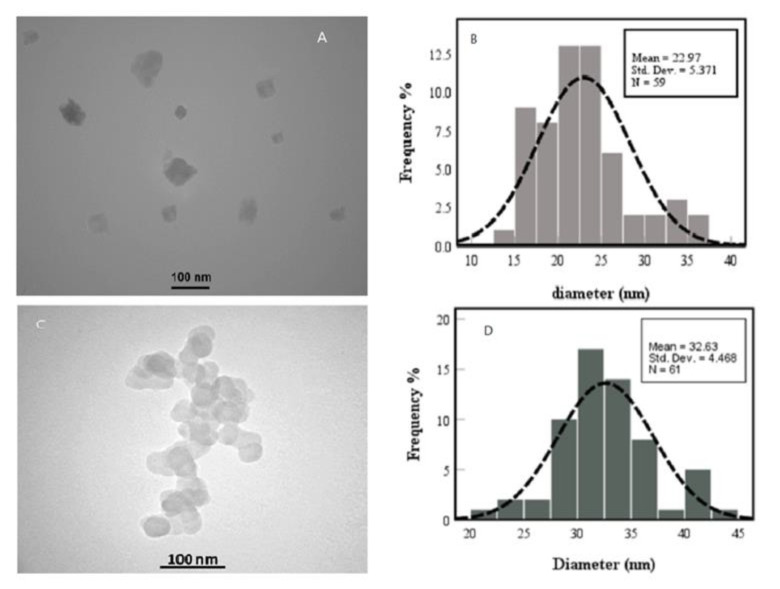
TEM images and size distribution of CNCs (A and B); and CNCs-TMX (C and D).

The TEM images are presented in Figure4A; TEM images display the spherical CNCs with an average diameter of 22.97±5.36 nm and signify their possession of a uniform morphology and size distribution. Furthermore, the observed spherical shape is indicative of a high aspect ratio that can influence the mechanical and chemical properties of this product. According to Figure4B, the addition of TMX to the CNCs significantly increased the diameter of CNCs to 32.63 ±4.46 nm as a sign of morphological changes caused by their interaction.

#### FE-SEM analysis

FE-SEM analysis has proven to be an effective tool for the characterization of CNCs due to its ability to provide high- resolution images with detailed information on the factors of size, shape, surface morphology, and size distribution. In addition, this technique can be also used to study the effects of different processing conditions on the structure and morphology of CNCs, which is valuable for understanding the properties and behavior of these materials.

**Figure 5:**
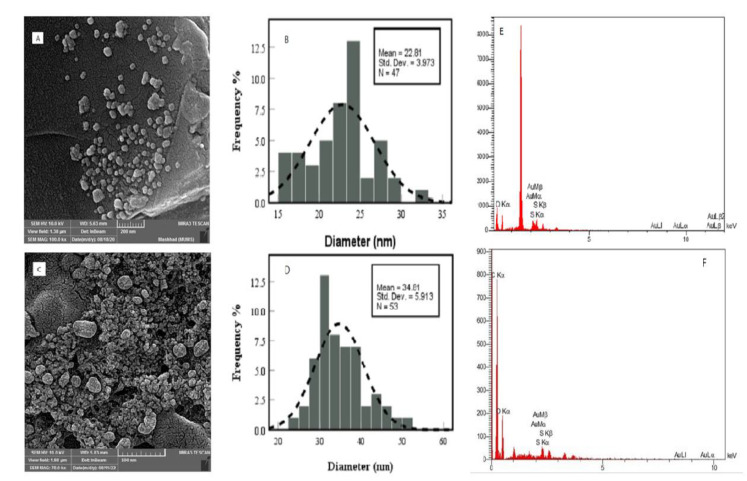
FE-SEM images and the average size of CNCs (A and B); CNCs-TMX (C and D); Energy- dispersive X-ray spectroscopy (EDX) diagram of CNCs (E), and CNC-TMX (F).

The rough surfaces of cellulose fibers are caused by their natural covering of non- cellulosic materials known as lignin and hemicellulose. However, these amorphous regions can be removed during the acid hydrolysis step of applying sulfuric acid. Based on FE-SEM images (Figure5A-B), the size of synthesized CNCs was increased to approximately 34.81±5.91 nm as a result ofchanges induced in the course of synthesizing process. Meanwhile, the next image exhibits the spherical shape of this product that had desirably remained unchanged upon the addition of TMX. According to the obtained EDX charts, the amount of C-O-S and Au elements for CNCs (Figure5C) were 25, 20, 4, and 29%, respectively, and the amount of C-O and Au elements for CNCs-TMX (Figure5D) were 71, 17, and 12%, respectively (Adil et al. 2020; Olad, Doustdar and Gharekhani 2020).

#### X-ray diffraction

**Figure 6:**
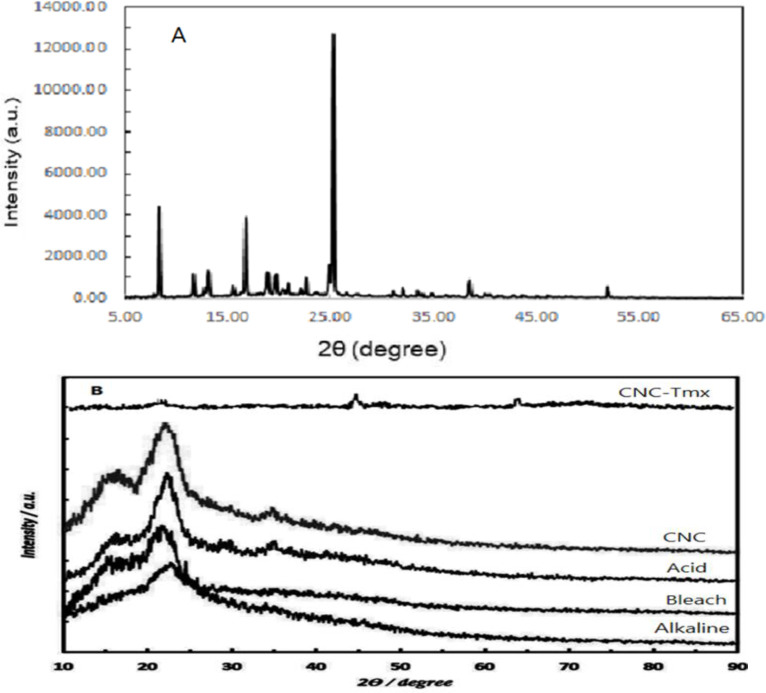
XRD pattern of TMX (A), XRD patterns of Alkaline treated, Bleach, Acid hydrolysed, CNC, and CNC-Tamoxifen (CNC-Tmx)(B).

The intensity and position of these peaks can provide data on the degree of crystallinity, as well as the possibly induced changes in crystal structure during the preparation of CNCs (Beigoli et al. 2022). X-ray diffraction diagrams of raw walnut shell fiber treated fiber by alkaline and bleaching method, acid hydrolysis, extracted cellulose nanocrystals, TMX, and TMX-loaded CNCs are presented in Figure 6. Cellulose is composed of a crystalline region, as well as an amorphous region that is consisted of hemicellulose and lignin. According to Figure 6, the detected two peaks in the XRD pattern (in 2θ = 15.5° and 2θ = 34.5°) were indicative of a decrease in lignin and hemicellulose and an increase in cellulose crystalline structure; this phenomenon was caused by the removal of lignin and hemicellulose subsequent to the chemical treatments. Since the presence of lignin and other impurities can affect the optical and mechanical properties of resulting CNCs, the main purpose of performing a bleaching process is to remove the residual lignin and other colored impurities from the raw materials. The purity percentage of formula below was 50%. I_002_ refers to the maximum intensity of diffraction peak from the crystalline region of cellulose approximately peak at 2θ = 22.5°, whereas I stands for the minimum intensity (between I_002_ and I_110_) from the amorphous region(Nasiri and Nasiri 2016; Zhou et al. 2018). Since the applied drug decreases the size and increases the purity percentage of the material, the CNCs-TMX chart shows a higher increase in the percentage of purity above this value, which thereby enhances the strength of the material by improving its crystallinity, moisture property, and hardness. Consequently, it becomes possible to produce nano-composites with advanced mechanical properties. However, the crystallinity of CNCs-TMX was decreased by the occurrence of bindings, which caused a reduction in the intensity as well. We detected the appearance of certain peaks in the higher 2θ such as 45° and 63.

### Fourier transform infrared spectroscopy

**Figure 7:**
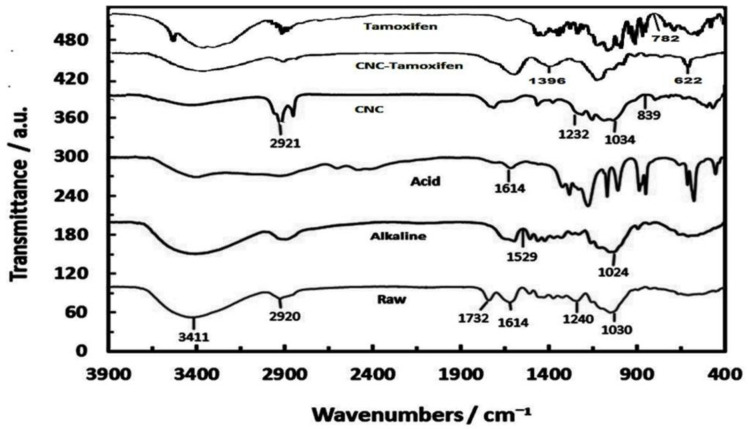
FTIR spectra of; Raw cellulose, Alkaline treated, Acid hydrolyzed, CNC, CNC- Tamoxifen, Tamoxifen.

The Figure 7 visually depicts the infrared diagram of walnut shell throughout different stages of treatment, including the raw walnut shell, alkaline, acid and CNCs, CNC-TMX, and TMX. The data of FTIR spectroscopy was gathered to differentiate and identify the existing active groups in each of the samples. A particularly intriguing finding in Figure 7 was the presence of 3411 cm-1peaks in the raw sample, which were attributed to the hydrophilic nature of samples and the stretching vibration of hydroxyl group. In comparison to the other samples, the interesting observance of a significant reduction in the nano-crystal band confirmed the successful removal of non-cellulosic components.

The close analysis of walnut skin fibers discovered the appearance of lignin and hemicellulose in the form of 1240 cm^-1^, 1614 cm^-1^, and 1732 cm^-1^ peaks, which were noticeably absent upon the synthesis of fibers. The peak situated at 1732 cm^-1^ in the raw walnut skin spectrum was attributed to the stretching vibration of acetyl and uronic ester group of hemicellulose or the ester bond of carboxylic groups of ferulic and coumaric acids, involving either lignin or hemicellulose. The absorption peaks in the spectral region of 1614 cm^-1^ to 2920 cm^-1^ throughout the untreated sample were caused by the presence of lignin and reflected the stretching of hydroxyl and carbon groups, which is clearly evident through the complete removal of lignin in the nano-crystal cellulose sample. The absorption peak at 1614 cm^-1^signified the stretching vibration of carbonyl group in the cellulose rings, as well as the bending vibration of hydroxyl group in the absorbed water molecule. This peak was decreased after facing different chemical treatments due to the loss of water samples. Meanwhile, the presence of this peak in raw walnut skin was related to the carboxyl bond. The other noteworthy peaks of 1034 cm^-1^ and 839 cm^-1^ were associated with the carboxyl stretching and vibration, as well as the purity of cellulose as one of the key characteristics of glycosidic bonds within cellulose. Overall, the findings were suggestive of the successful removal of lignin and hemicellulose through different chemical treatments, resulting in the production of high-quality nano-crystalline cellulose. Most of the non-cellulosic components were removed during the hydrolysis and bleaching process.

The absorption at 1240 cm^-1^ can be attributed to the bending frequencies, whereas the detected peak at 1232 cm^-1^was drastically reduced upon the loss of hemicellulose materials. In addition, the absorption spectrum at 1614 cm^-1^ and 1529 cm^-1^ could be associated with the alteration of water angles. These two peaks are easily noticed in both the untreated and acidified fibers as an impact of cellulose higher exposure on intensifying the hydrophilic state of surrounding materials. This absorption spectrum can also be linked to the stretching of carbon double bonds in aromatic rings, which can be connected to lignin. Chemical processes can be used to remove non-cellulosic materials due to the existence of a significant number of these groups in the chemical structure of lignin. However, the recorded peak at 1732 cm^-1^is related to the carboxyl bond that is typically found in the amorphous bonds of aliphatic esters caused by carbonyl stretching in hemicellulose and lignin. The detected peaks around the region of 2925-3411 cm^-1^ can be linked to both asymmetric and symmetric stretching and bending vibrations. Moreover, COH bending, symmetric vibrational bonds connected to C-O-SO3, CH2 symmetric bending, and OH bending were presented through the peaks of 622 cm^-1^, 782 cm^-1^ and 1396 cm^-1^, respectively.

### Loading capacity and encapsulation efficiency measurement

The LC and EE values were calculated to be 27.48 ± 1.16 and 86.22 ± 1.42, respectively. These satisfactory results prove the effectiveness of cellulose nanocrystals in improving the bioavailability of TMX.

### Evaluation of the binding behavior between TMX and CNCs

#### Fluorescence spectroscopy measurements

**Figure 8:**
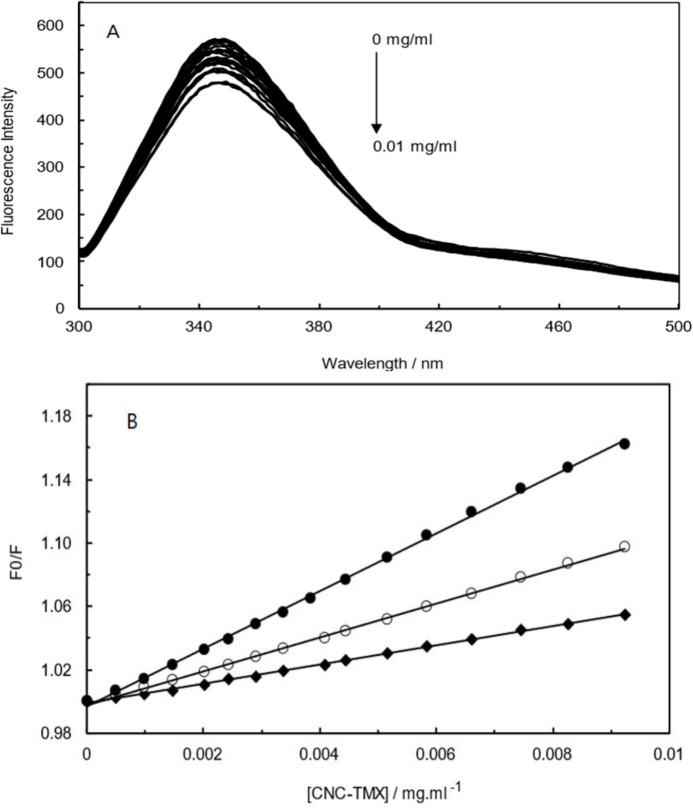
(A); Fluorescence emission spectra of Hb (CNCs-TMX) with various amounts of CNCs-TMX (0-0.1 mg/ml) at pH=7.4, T=298 K, λex=280 nm. (B); Stern-Volmer plots of Hb (CNCs-TMX) at different temperatures (298, 303 and 308K).

Figure 8A presents the fluorescence spectra of Hb upon the gradual addition of CNCs-TMX. According to this diagram, the fluorescence intensity of Hb complex was decreased as a result of increasing the concentration of CNCs-TMX. Fluorescence quenching can be classified into the main two types of static and dynamic quenching. Static quenching process implicates the formation of a non- fluorescent complex by the fluorophore and quencher that results in decreasing the fluorescence intensity, while dynamic quenching occurs by the collisional interaction of fluorophore with quencher. Stern-Volmer equation is used to distinguish the dominant type of these two quenching (Gehlen 2020):

F0/F=1+kq τ0 [Q] = 1+Ksv[Q] (3)

In this equation, F0 and F stand for fluorescence intensity in the absence and presence of [Q], Ksv represents the Stern- Volmer quenching constant, kq is the quenching rate constant, τ0 (10^-8^ s) refers to the normal fluorescence lifetime without a quencher, and [Q] presents the quencher concentration. Figure8B displays the Stern-Volmer plot of 𝐹_0_⁄𝐹 against quencher concentration at three different temperatures (298, 303, and 308 K) and pH = 7.4, while the obtained Ksv values are listed in Table 1. These data indicated the negative correlation of Ksv values with temperature, which is generally known as an indication of static quenching predominance(Jiang et al. 2020).

### Synchronous fluorescence spectroscopy (SFS)

**Figure 9:**
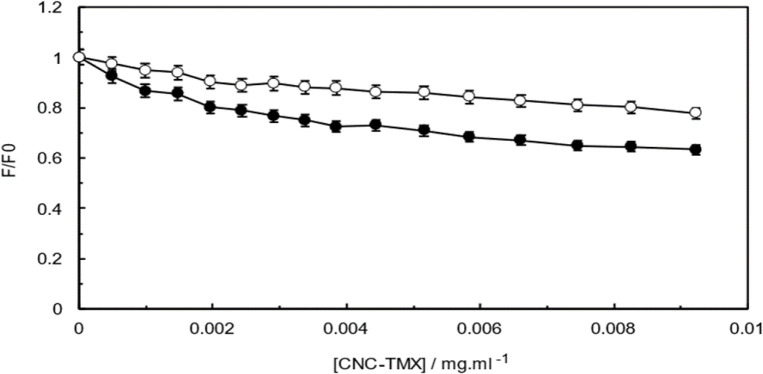
Synchronous fluorescence spectra of Hb with various concentrations of CNCs-TMX at ∆λ=15 nm (open circles) and ∆λ=60 nm (closed circles) T=298 K, pH=7.4.

The powerful tool of SFS in ligand- protein interaction studies can facilitate the simultaneous monitoring of excitation and emission wavelength for achieving a better understanding over the conformational changes induced in the protein(Sunuwar and Manzanares 2021). Therefore, the fluorescence emission spectra of Hb in the presence of different concentrations of CNCs-TMX were recorded at two simultaneous wavelengths (∆λ=60 nm and ∆λ=15 nm) and the results were included in Figure 9. The plot of molar ratio displays a higher slope upon the wavelength setting of ∆λ=60 nm when compared to ∆λ=15 nm, which reveals the considerable contribution of Trp residues in the interaction.

### Circular dichroism (CD) measurements

**Figure 10:**
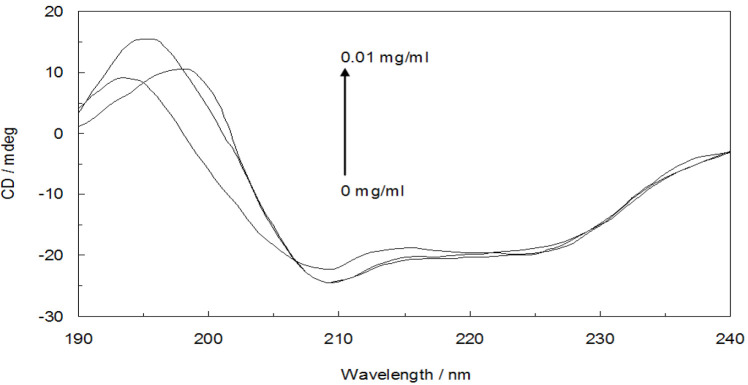
Far-UV CD spectra of Hb in the presence of various concentrations of CNCs-TMX (0-0.01 mg/ml), [Hb]= 0.03 % w/v, T=298K, pH=7.4.

Figure10 depicts the results of recorded Far-UV CD spectre in the presence and absence of different concentrations of the complex (CNCs-TMX) at pH = 7.4 and room temperature. The accommodation of two minimums in the 208 and 222 nm points of Far-UV region can signify the α- helical structure of a protein. In conformity to the listed values for different motives of secondary structure in Table 2, the secondary structure of protein was evidently changed upon the addition of CNCs-TMX. More specifically, α-helix content and β-sheets were slightly decreased and faced the following enhancement in random coils as a further approve on the slight unfolding of protein.

### Resonance light scattering (RLS)

**Figure 11:**
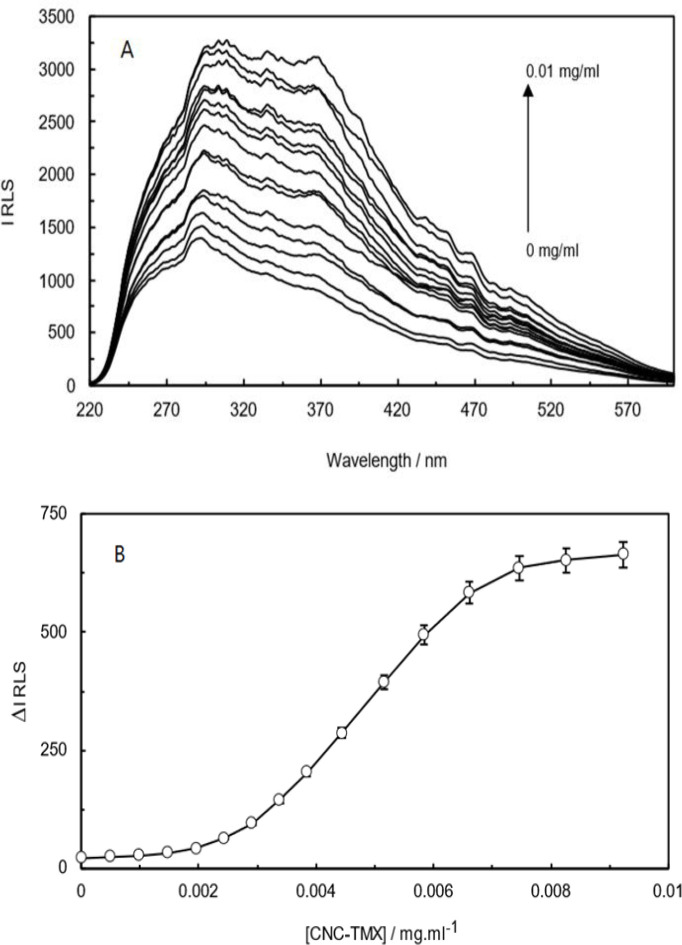
RLS spectra of Hb (CNCs-TMX) complex (A), and the curve of ∆IRLS of Hb in the presence of different concentrations of CNCs-TMX at T=298 K, pH=7.4 (B).

As depicted in Figure11A, the obtained spectrum after the addition of CNCs-TMX to Hb solution exhibited an increase in the intensity of resonance light scattering. This observation can be interpreted as the complex behavior for protracting the moles number of components in the environment, promoting the binding of ligand to Hb, and increasing the size of protein. The magnitude of resonance light-scattering increment highlights a deviation from the spherical state of particles, while indicating the presence of a strong maximum and sharp peak at 295 nm in contrast to the recorded broad band at around 370 nm.

### Fluorescence resonance energy transfer (FRET)

The performance of this technique (FRET) requires the preparation of three essential criteria. Firstly, the absorbance spectrum of acceptor molecule must overlap with the emission spectrum of donor molecule. Secondly, the distance between the donor and acceptor molecules should be in the range of 2-8 nm and thirdly, the orientation of the transition dipole of acceptor and donor molecules mustbe parallel, or in other words, approximately aligned. The facilitation of these criteria provides the successful application of FRET for researchers in order to obtain critical information on molecular interactions, such as determining the distances between molecules in a solution, monitoring conformational changes, and identifying the changes of protein-protein or protein-ligand interactions (Wu et al. 2020).

**Figure 12:**
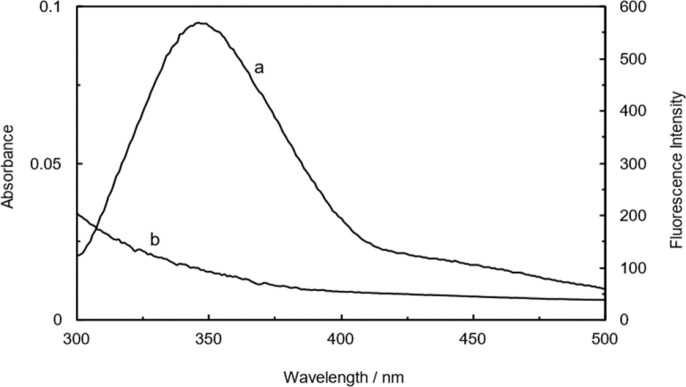
Overlap of the fluorescence emission spectrum of Hb (curve a) with the absorbance spectrum of CNCs-TMX (curve b). [Hb]=[CNCs-TMX] =0.03 mg/ml, T=298 K, pH=7.4.

Figure 12 exhibits the satisfactory overlap of emission and absorption spectra. Moreover, the obtained values (Table 3) indicated the possibility of transferring energy from the protein (donor) to CNCs- TMX (acceptor) and confirmed the occurrence of interactions between the Hb protein and TMX-loaded CNCs (Makarska- Bialokoz and Lipke 2019).

### Three-dimensional fluorescence spectra studies

**Figure 13:**
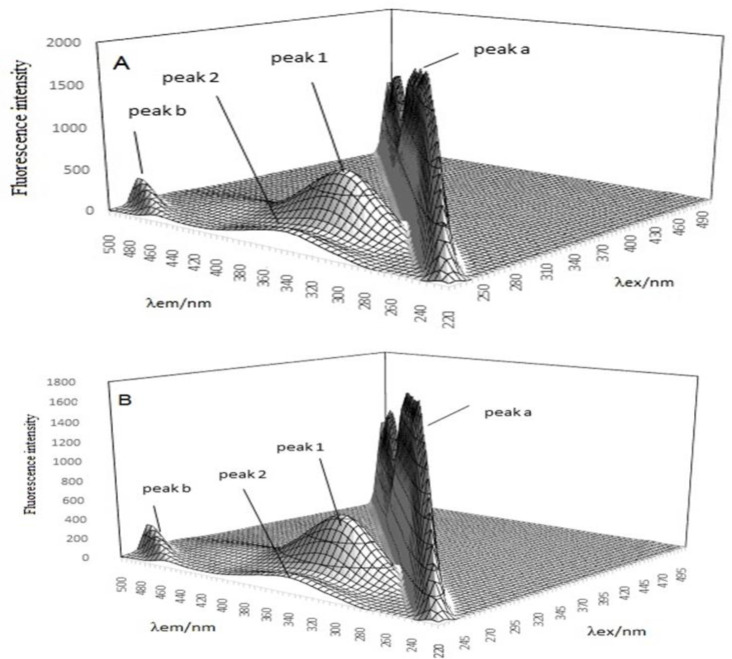
Three-dimensional fluorescence of Hb (A), and Hb (CNCs-TMX) (B).

The displayed peak 1 (λex = 275, λem= 330) in Figure 13A, represents the spectral behavior of Tyr and Trp residues of Hb protein, while peak 2 stands for the π→π* transition of the backbone structure of polypeptide. Peak a is expressive of RLS as λex equals to λem and peak b (λem= 2λex) refers to second-order scattering. In Figure 13B, the observed enhancement in the peak of fluorescence intensity (a) and a reduction in peak b,1,2 confirmed the microenvironmental changes of protein and proved the efficient interaction of ligands with protein. These results are consistent with the data of RLS(Glatzel and Bergmann 2005).

### Oxygen-Hemoglobin dissociation analysis

**Figure 14:**
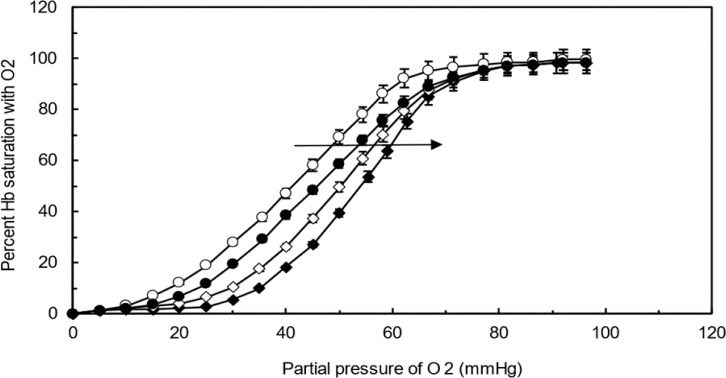
Oxygen-Hemoglobin Dissociation Curve (ODC) in the presence of CNCs-TMX complex.

In conformity to the presented ODC curve at the presence of CNCs-TMX in Figure 14, the generally sigmoidal shape of the curve expresses the impact of increasing the partial pressure of oxygen on intensifying the Hb saturation with oxygen up to the point of reaching a plateu, where the Hb becomes fully saturated by oxygen. This figure also displays the shifting of ODC curve to the right at the presence of TMX-loaded CNCs, which may be attributed to the binding of TMX to hemoglobin. Apparently, this binding enhances the release of oxygen from hemoglobin molecules(Chu et al. 2020). In other words, the interaction of CNCs-TMX with Hb can reduce the affinity of hemoglobin for oxygen and pave the way for the more readily release of oxygen into tissues at a given oxygen pressure. Consequently, CNCs-TMX binding causes hemoglobin to be less saturated with oxygen for any given partial pressure of oxygen.

### Effect of CNCs-TMX on cell viability

**Figure 15:**
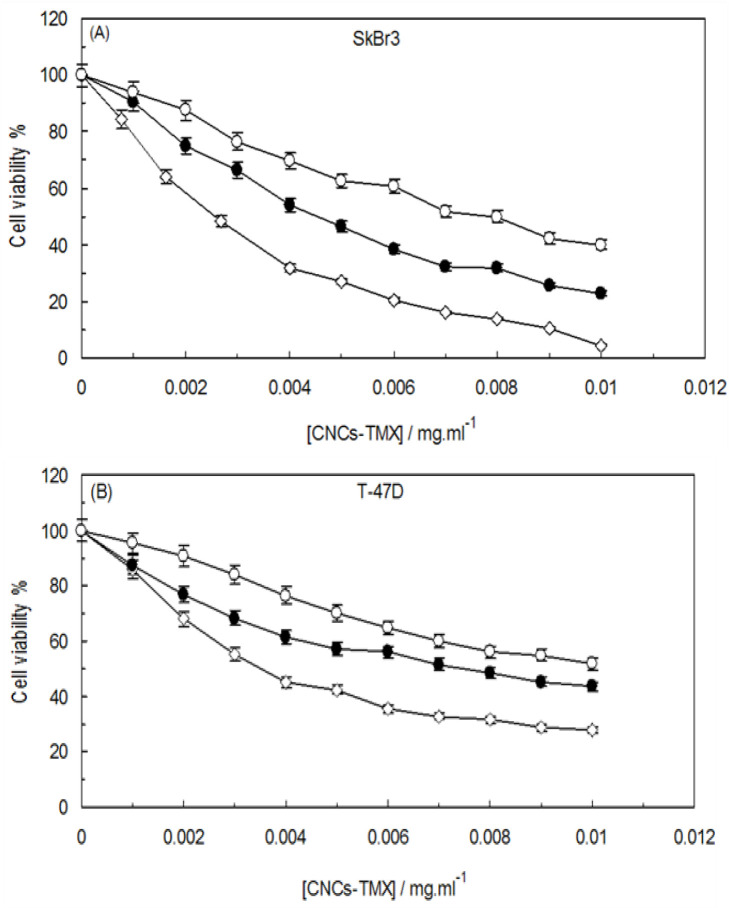
Effects of CNCs-TMX on the cell viability of (A); SkBr3 cell line, (B); T-47D cell line. SkBr3 and T-47D cells were treated with different concentrations of CNCs-TMX for 24 hr (Open Circles), 48 hr (Closed Circles) or 72 hr (Open Diamonds).

The cytotoxic potential of CNCs-TMX was assessed by the application of MTT assay, for which SkBr3 and T-47D cells were exposed to varying concentrations of CNCs-TMX (ranging from 0.002 to 0.01 mg/mL) for different time intervals (24, 48, and 72 hr). As illustrated in Figure 15 (A, B), CNCs-TMX elicited a dose-dependent reduction in the viability of these cells. The treatment of cancer cells with low concentrations of CNCs-TMX (<0.002 mg/mL) for 24-72 hours did not cause any signifiant changes in the declining trend of cell viability. However, the viability of SkBr3 and T-47D cells remained above 40% at a concentration of 0.006 mg/mL, even after 72 hours of CNCs-TMX treatment. Notably, a significant decrease in the rate of viability was observed in both SkBr3 and T-47D cells as the applied concentration of CNCs-TMX surpassed 0.006 mg/mL.

### Effect of CNCs-TMX on the migration and invasion of SkBr3/ T-47D cells

**Figure 16:**
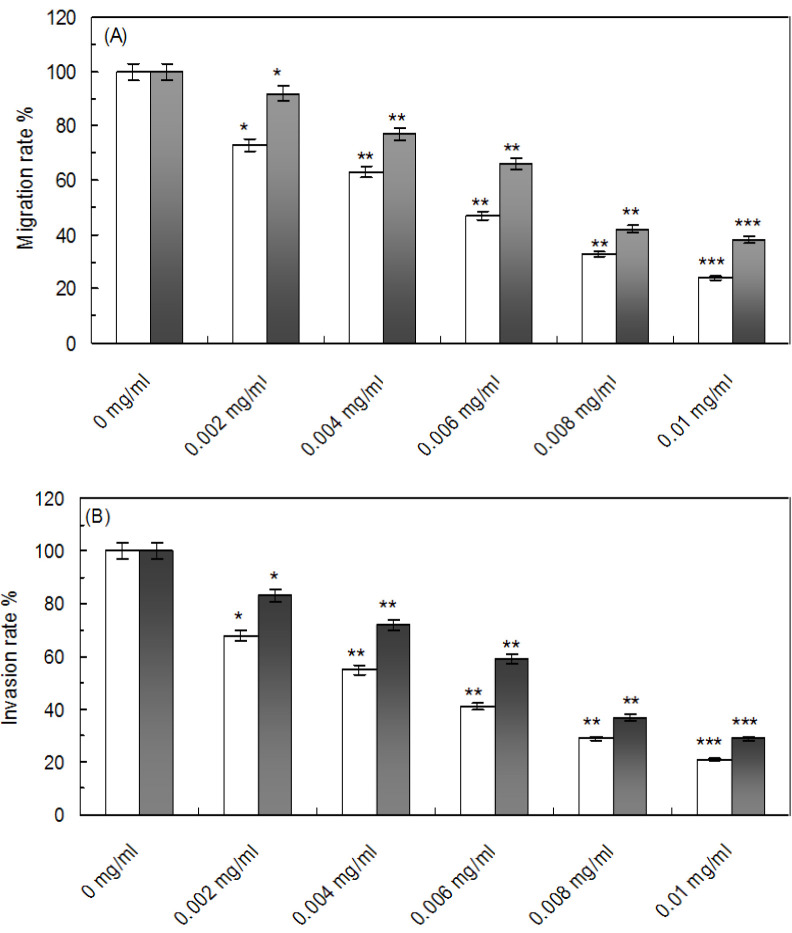
Effects on the migration and invasion of SkBr3/T-47D cells caused by CNCs- TMX. (A); white columns (SkBr3 cell line), Cells that invaded through the membrane were quantified under different concentrations of CNCs-TMX. (B); Grayscale columns (T-47D cell line) Migrated cells were quantified by manual counting under different concentrations of CNCs-TMX. Data was analyzed by the mean SEM (n = 3) as *p<0.05, **p<0.01 and ***p<0.001 in comparison with the control.

A wound healing assay was exerted to evaluate the impact of CNCs-TMX on the migration of SkBr3/T-47D cells, which exhibited a concentration-dependent inhibitory effect on the migration of both cells as it is depicted in Figure 16A. Notably, the superior effectiveness of CNCs-TMX in inhibiting the migration of SkBr3 cells was indicated by observing a comparatively lower migration rate than the case of T-47D cells. Moreover, we performed transwell assays to further investigate the effect of CNCs-TMX on the factor of invasion. In conformity to Figure 16B, the observance of a decrease in the migration rates of cells subsequent to enlarging the concentrations of CNCs- TMX indicated that the application of higher volumes led to the stronger inhibition of cell migration in both SkBr3 and T-47D cells. Moreover, this dose- dependent effect was more pronounced in SkBr3 cells as compared to that of T-47D cells.

**Figure 17:**
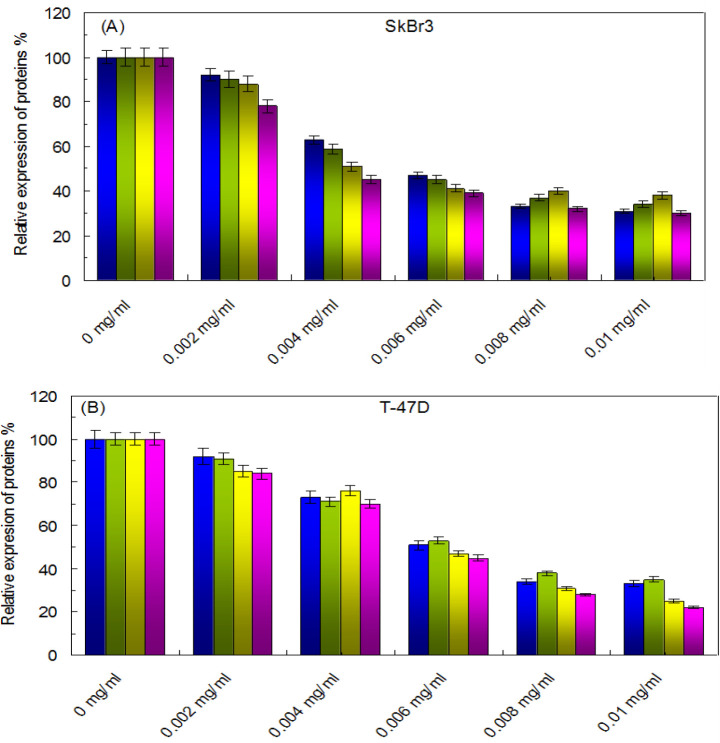
Effects on the expressive levels of pivotal proteins of RAS/RAF/MEK/MAPK signaling pathway in SkBr3/T-47D cells caused by CNCs- TMX. (A, B); The expressions of Ras (blue), Raf (green), MEK (yellow) and MAPK (pink) in SkBr3/T-47D cells under different concentrations of CNCs-TMX.

Figure 17A depicts the dose-dependent decrease of RAS, RAF, MEK, and MAPK expression levels in SkBr3 cells as a result of increasing the applied concentrations of CNCs-TMX. However, in regards to T-47D cells, the expression of these proteins faceda comparatively lower extent of reduction as it is displayed in Figure 17B. These results indicated the inhibiting effect of CNCs-TMX on RAS/RAF/MEK/MAPK signaling pathways by down-regulating the expression of proteins associated with cancer metastasis.

### Release behavior of TMX

**Figure 18:**
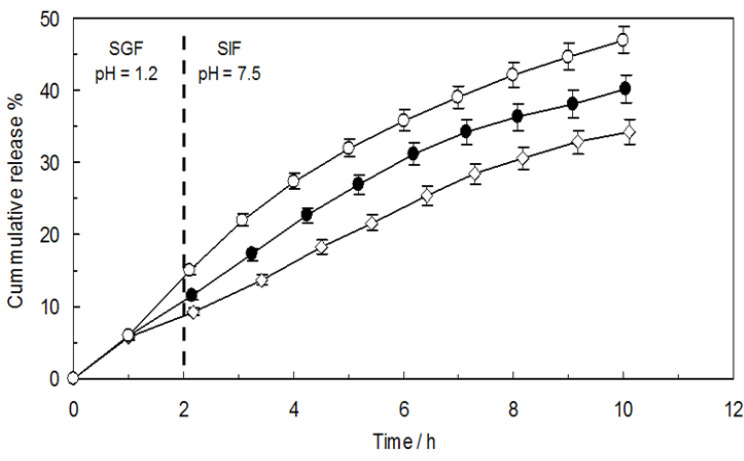
Release profile of TMX from CNCs-TMX (Open diamonds), TMX-loaded PEG-PPG-PEG micelles (Closed circles) and TMX-loaded niosomes (Open circles).

Drug delivery systems with a controlled releasing manner are evidently designed to ensure the release of entrapped drug in specified amounts over a defined period of time(Fan et al. 2022). The in vitro release behavior of TMX-containing samples, labeled as CNCs-TMX, TMX-loaded PEG- PPG-PEG, and TMX-loaded niosomes, were characterized by the usage of successive simulated gastrointestinal digestion. According to Figure 18, after 2 hr of gastric digestion in the presence of pepsin, the percentages of cumulative TMX release from CNCs-TMX, TMX-loaded PEG-PPG-PEG, and TMX-loaded niosomes were 9.3%, 11.6% and 15%, respectively, and also after 6 hr of digestion (2 hr in SGF with 4 hr in SIF), the released percentages were calculated to be 25.4%, 31.1% and 35.8%, respectively. The outcomes indicated the remarkably slower release of encapsulated TMX in CNCs- TMX than that of the TMX-loaded PEG- PPG-PEG and TMX-loaded niosomes in both SGF and SIF environments during the release experiment.

## Discussion

The size distribution of particles is determined by analyzing the rate of detected fluctuation in the scattered light. The technique of DLS is used to measure the sizes of particles ranging from a few nanometeres to several micrometeres. A high value of zeta-potential (either positive or negative) is the sign of a strong repulsion between particles that prevents them from coming into close contact and results in an unstable suspension. Conversely, a low zeta-potential value signifies the existence of a weak repulsion or attraction between the particles that leads to their aggregation and flocculation. The observed increase in DLS upon the addition of TMX to CNCs solution could be attributed to the reduced electrostatic repulsion between particles that caused the formation of larger aggregates and ultimately increased the rate of size distribution. Furthermore, the zeta- potential of a solution could be also increased subsequent to the addition of TMX due to the induction of charge compensation. TMX can effectively counterbalance the negative charge of CNCs surface by adsorbing onto the surface of cellulose nanocrysals and consequently increase the zeta-potential of solution, which is recognized as a stabilization effect.

The observation from this technique may be also attributed to effect of TMX adsorption onto the surface of CNCs on increasing the interfacial area between the CNCs and surrounding media, which there by extended the diameter of CNCs particles. The walnut shell nanocrystals were obtained by treating the walnut shell with alkaline and acid hydrolysis to remove its impurities such as hemicelluloses and lignin(Neto et al. 2013).

The two main peaks observed in CNCs XRD patterns at the 2θ angles of around 22- 23° and 34-35° could be related to the crystalline structure and amorphous regions, respectively. The peak in 2θ =22.5° throughout the X-ray diffraction pattern represents the crystallinity index of our samples, while the observance of this peak is a requirement for the diagnosis of crystal structures. The sharper state of this peak in the pattern of chemically treated CNCs, when compared to other samples, indicated the removal of amorphous regions and the significant enhancement of CNCs crystalline structure.

By comparing the spectra of untreated raw fibers and hydrolyzed particles and CNCs, CNCs-TMX, and TMX, the peaks were observed to become smaller and even sometimes disappear during the synthesizing process. The disappearance of these peaks in the spectrum of nanocellulose shows the total removal of lignin and hemicellulose through different stages of extraction process. Also, as a result of adding drugs to CNCs, the peaks faced major alterations and became smaller that is a sign of structural changes. Also, this observation approves the bindings occurrence and the drugs attachment to CNCs (Maaloul et al. 2021).

Loading Capacity (LC) and Encapsulation Efficiency (EE) refers to the proportion of loaded TMX that is effectively encapsulated within the CNCs matrix. The actual amount of exerted TMX for creating the drug-CNCs complex, as opposed to being lost in the process, can be determined by this factor. Therefore, a high loading capacity refers to the possibility of attaching a large amount of TMX to each CNCs molecule, while a high EE indicates the effective encapsulation of a high proportion of loaded TMX within the CNCs matrix, which ensures the availability of a large concentration of the drug for release over time.

Fluorescence measurements were involved to analyze the binding behavior in the formation of Hb (CNCs-TMX) complex. Fluorescence spectroscopy measurements were carried out to assess the impact of TMX-loaded CNCs binding to the structure of Hb.

This measurement functions by the usage of bands with distinct shapes and magnitudes in the Far-UV range and is exerted to obtain a clear image of how the complex (CNCs-TMX) affects the secondary structure of human Hb such as α- helix, β-sheets, β-turns, and random coil structures. Considering these facts and the close relationship of proteins functionality with their secondary structure, it can be concluded that the interaction between CNCs-TMX complex and Hb led to the inducement of conformational changes in Hb(Pacheco et al. 2022).

In the current study, Hb was introduced to the device's cell and the system was configured to ∆λ = 0. The experimental setup included the irradiation of a beam and the measurement of emitted beam with a value equal to 0 for inducing resonance light scattering. These observations added a significant extension to the scope of performed studies on the characteristics of particles in a solution, specifically those relating to particle size and aggregation, leading to the identification of potential applications in a wide range of fields. The main advantage of this technique is the determination of three outputs, including λem, λex, and fluorescence intensity, to provide a more accurate and trustable 3D fluorescence spectroscopy than a typical fluorescence spectroscopy.

Due to the significance of this process in delivering oxygen to tissues throughout the body, any abnormalities in oxygen- hemoglobin dissociation can result in a wide range of pathophysiological conditions. In this regard, we considered the utilization of ODC curve in order to study the effect of TMX-loaded CNCs on oxygen-hemoglobin dissociation (Collins et al. 2015).

Through the implication of two representative breast cancer cell lines, our study demonstrated the effective inhibiting power of CNCs-TMX towards the proliferation, migration, and invasion of breast cancer cells by targeting the key proteins of RAS/RAF/MEK/MAPK pathways. It is vital to consider the dependency of these effects on culturing time and drug dosage in order to provide aoretical basis for potential clinical applications. Our findings highlight the promising stance of CNCs-TMX as a therapeutic agent for suppressing breast cancer metastasis. Our data are similar to the work of Ruma Majiet al. 2014(Maji et al. 2014), which employed Tamoxifen citrate loaded polylactide-co-glycolide (PLGA) based nanoparticlesas a TMX delivery system and reported the prolonged release of sample in simulated intestinal fluid.

The extraction of CNCs from walnut shell was successfully executed through the application of chemical and mechanical treatments under the objective of designing an effective platform for the delivery of TMX. The synthesized CNCs contained a fine colloidal stability and acceptable crystallinity index. Despite the spherical shape of both samples, an increase was observed in the size of CNCs-TMX sample. The obtained data can safely confirm the reliable functionality of produced CNCs as a carrier for TMX, which can be used in the treatment of breast cancer to speed up its process and reduce the induction of side effects by drugs. Furthermore, we studied the interaction behavior of CNCs-TMX and human Hb through various biophysical methods and realized the dominance of static quenching, as well as observed the efficient binding of CNCs-TMX to Hb protein. The observance of a 7 nm distance between the Hb and (CNCs-TMX) was indicative of a successful interaction. In coordination with the oxygen-hemoglobin dissociation curve, the binding of CNCs- TMX to Hb caused a reduction in the affinity of Hb to oxygen and consequently enhanced the release of oxygen from Hb. In this study, we demonstrated the capability of CNCs-TMX to inhibit the proliferation, migration, and invasion of breast cancer cells by down-regulating the expressions of RAS, RAF, MEK and MAPK. The results of release test under simulated gastrointestinal conditions were suggestive of a controlled manner throughout the release of TMX from CNCs-TMX.

**Table 1:** K_sv_ values of Hb (CNCs-TMX) complex at three different temperatures.

**T / K**	**K** **sv ** **/ L.gr** ^-1^
298	(1.82 ± 0.06) × 10^4^
303	(1.06 ± 0.08) × 10^4^
308	(0.61 ± 0.07) × 10^4^

**Table 2:** The fraction of the secondary structure of Hb in the absence and presence of CNC_s-_ TMX at two different concentrations.

**System**	α**-helix %**	β**pleated-Sheet %**	**Turn %**	**Unordered Coil %**
Hb	46.12 ± 0.07	29.63 ± 0.07	12.35 ± 0.07	11.9 ± 0.07
Hb (CNCs-TMX) 0.005 mg.ml^-1^	45.92 ± 0.09	29.54 ± 0.09	12.31 ± 0.09	12.23 ± 0.09
Hb (CNCs-TMX) 0.01 mg.ml^-1^	45.55 ± 0.08	29.46 ± 0.08	12.27 ± 0.08	12.72 ± 0.09

**Table 3:** The distance of Trp for Hb in the presence of CNCs-TMX.

System	J / cm3. L. mol^-1^	E	R_0_ / nm	r / nm
Hb (CNCs-TMX)	6.23 × 10^-15^	0.1	3.5	2.17
